# Cuprous Halide Coordination Polymer for Efficient NIR-I Photothermal Effect and Photo-Thermo-Electric Conversion

**DOI:** 10.3390/molecules29246034

**Published:** 2024-12-21

**Authors:** Ning-Ning Zhang, Xiang-Tong Liu, Ke Xu, Ya-Tong Liu, Lin-Xu Liu, Yong Yan

**Affiliations:** School of Chemistry and Chemical Engineering, Liaocheng University, Liaocheng 252000, China; 13583374079@163.com (X.-T.L.); zhuangzhang0653@163.com (K.X.); 15610092260@163.com (Y.-T.L.); l18554696997@163.com (L.-X.L.)

**Keywords:** cuprous halide coordination polymers, black photothermal agent, near-infrared photothermal effects, photo-thermo-electric conversion

## Abstract

Photo-thermo-electric conversion devices represent a promising technology for converting solar energy into electrical energy. Photothermal materials, as a critical component, play a significant role in efficient conversion from solar energy into thermal energy and subsequently electrical energy, thereby directly influencing the overall system’s efficiency in solar energy utilization. However, the application of single-component photothermal materials in photo-thermo-electric conversion systems remains limited. The exploration of novel photothermal materials with broad-spectrum absorption, a high photothermal conversion efficiency (PCE), and a robust output power density is highly desired. In this study, we investigated a black cuprous halide compound, [Cu_2_Cl_2_PA]**_n_** (**1**, **PA** = phenazine), which exhibits broad-spectrum absorption extending into the near-infrared (NIR) region. Compound **1** demonstrated a high NIR-I PCE of 50% under irradiation with an 808 nm laser, attributed to the metal-to-ligand charge transfer (MLCT) from the Cu(I) to the **PA** ligands and the strong intermolecular π–π interactions among the **PA** ligands. Furthermore, the photo-thermo-electric conversion device constructed using compound **1** achieved a notable output voltage of 261 mV and an output power density of 0.92 W/m^2^ under the 1 Sun (1000 W/m^2^) xenon lamp.

## 1. Introduction

Solar energy, as a ubiquitous and abundant green energy resource, offers significant potential to reduce pollution, mitigate global warming, and alleviate the pressures arising from fossil fuel scarcity [1,2]. Among the various solar energy utilization technologies, photo-thermo-electric conversion has emerged as a promising approach for directly converting solar energy into electrical energy, garnering substantial attention [3]. This technology relies on the synergistic interplay between two fundamental energy conversion processes: photothermal conversion and thermo-electric conversion based on the Seebeck effect [4]. Upon solar irradiation, photothermal materials convert light energy into thermal energy, creating a temperature difference (ΔT) across the thermo-electric device. This temperature gradient drives the generation of a potential difference within the thermo-electric device, enabling the production of electrical energy. Photo-thermo-electric conversion devices offer several advantages, including a wide response wavelength range, self-sustainability, miniaturization, and portability [5]. These attributes make them particularly suitable for applications such as outdoor power generation, remote area power supplies, wearable electronics, and space exploration [6].

Photothermal materials, as a critical component of photo-thermo-electric conversion devices, play a pivotal role in enabling the efficient transformation of solar energy into thermal energy and subsequently into electrical energy, directly impacting the overall system’s efficiency in solar energy utilization. Among the reported photo-thermo-electric conversion devices, the employed photothermal materials predominantly consist of composite nanomaterials based on metals [7,8,9], carbon [10,11,12], and polymers [13]. While these materials hold promise, their applications are hindered by limitations such as a low output voltage and power density (Table S1). Recently, single-component, broad-spectrum-absorption photothermal materials, including donor–acceptor (D-A) organic co-crystals [6], D-A organic molecules [14], and inorganic–organic hybrids [15,16], have started to gain attention in the context of photo-thermo-electric conversion. Notably, the output power densities of these single-component materials are generally higher than those of composite materials (Table S1). However, the application of single-component photothermal materials in this field remains limited to a few examples. Therefore, the development of novel single-component photothermal materials with broad-spectrum absorption, a high photothermal conversion efficiency (PCE), and a robust output power density continues to be a significant challenge.

Photothermal conversion is essentially a non-radiative transition process from an excited state to a ground state [17]. Hence, numerous strategies that affect this photophysical process can enhance the PCE of the compound, such as intermolecular π–π interactions [18], molecular motion [19], charge transfer [20], fluorescence resonance energy transfer [21], photoinduced electron transfer [22], and the heavy atom effect [23]. Among these, the formation of charge transfer states has been particularly attractive, as it not only broadens optical absorption but also facilitates the non-radiative decay of excited states. To date, many efficient photothermal materials with broad-spectrum absorption have been developed by realizing low-energy charge transfer states in organic systems [24,25]. In fact, in addition to organic systems, low-energy charge transfer states are also prevalent in organic–inorganic hybrids [26], particularly in cuprous complexes [27]. According to previous studies [28], Cu(I) complexes usually form metal-to-ligand charge transfer (MLCT) states when they are coordinated with electron-deficient ligands under light irradiation, and their optical features can be tailored by refining the molecular structure and energy levels of the ligands. Inspired by these characteristics, we selected a cuprous halide coordination polymer (**1**, [Cu_2_Cl_2_PA]**_n_**, **PA** = phenazine, CCDC no. 1175339) [29] from the Cambridge Crystallographic Data Centre (CCDC) database as the research object in this work. Compound **1** is black in appearance and exhibits a broad optical absorption band extending into the near-infrared (NIR) region. The experimental results revealed that compound **1** achieves a high NIR-I photothermal conversion efficiency (50%) under 808 nm laser irradiation, attributed to the MLCT from the Cu(I) to the **PA** ligands and the strong intermolecular π-π interactions among the **PA** ligands. Furthermore, a photo-thermal-electric conversion device based on compound 1 demonstrated excellent performance, achieving an output voltage of 261 mV and a power density of 0.92 W/m^2^ under 1 Sun (1000 W/m^2^) illumination.

## 2. Results and Discussion

**Figure 1:**
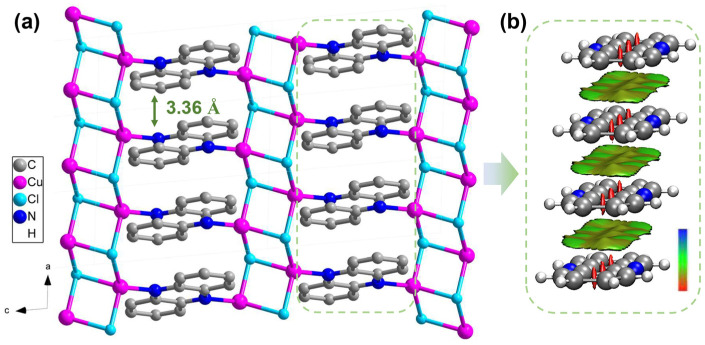
(**a**) The 2D layer structure of compound **1** as viewed along the *b* axis. (**b**) A selected π–π stacking fragment within compound **1**, along with its calculated gradient isosurfaces (s = 0.5 a.u.). The surfaces are colored using a blue–green–red (BGR) scale based on the values of sign (λ_2_)*ρ*, which range from −0.04 to 0.02 a.u. In this scale, blue indicates strong attractive interactions, green represents moderate attractive interactions, and red signifies strong non-bonded overlaps.

Compound **1** was synthesized using a novel solvothermal one-step method, which is simple and efficient and yields a high quantity of product (detailed procedures are provided in the Supporting Information). This approach offers significant advantages over the previously reported method, which required synthesis under an argon atmosphere, making it more complex and time-consuming [29]. The phase purity of compound **1** was confirmed using powder X-ray diffraction (PXRD) (Figure S1) and IR (Figure S2) spectroscopy. Additionally, a thermogravimetric (TG) analysis revealed that the crystal sample of compound **1** exhibits good thermal stability, with no obvious weight loss observed before 210 °C (Figure S3). The single-crystal X-ray diffraction (SXRD) analysis revealed that compound **1** crystallizes in the space group P-1. The detailed crystallographic data are summarized in Table S2. Each Cu(I) atom is fourfold-coordinated by three Cl atoms and one nitrogen atom from a PA ligand. Each Cl atom binds three Cu(I) atoms via *μ*3 coordination, resulting in a 1D cuprous chloride chain. The PA ligands bridge these chains by coordinating with Cu(I) atoms through their nitrogen atoms, resulting in a 2D network (Figure 1a). Furthermore, the PA ligands are tightly stacked, with an interplanar spacing of 3.36 Å (Figure 1a), contributing to the stability of the overall structure.

**Figure 2:**
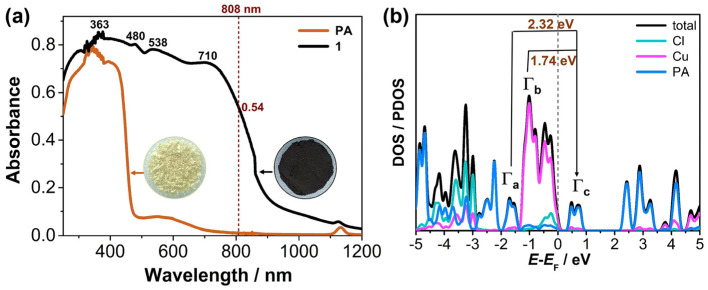
(**a**) Solid−state absorption spectra of **PA** and compound **1**. (**b**) TDOS and PDOS of **1**, with the Fermi level (*E*_F_) set to zero as a reference.

Interestingly, the crystalline powder sample of compound **1** appears black under ambient conditions (Figure 2a). Solid-state UV-Vis-NIR spectra reveal that compound **1** exhibits a broad optical absorption band spanning from the ultraviolet to the near-infrared region, with an absorption edge extending to 1200 nm, in contrast to the ligand **PA** (Figure 2a). To investigate the origin of this broad absorption spectrum, the absorption peaks were analyzed and assigned. The absorption peaks at 363 nm and 480 nm were attributed to the intrinsic absorption of the **PA** ligand. Density of states (DOS) and partial DOS (PDOS) calculations indicated that the Γ_a_ and Γ_c_ states primarily arise from the mixed antibonding *p_π_* orbitals of the **PA** ligands, while the Γ_b_ states originate from the *d* orbitals of Cu(I) (Figure 2b and Figure S4). As a result, the absorption peak at 538 nm (2.3 eV) was attributed to the transfer from the Γ_a_ state to the Γ_c_ state, corresponding to the ligand-to-ligand charge transfer (LLCT) among the **PA** ligands. The absorption peak at 710 nm (1.746 eV) was attributed to the transfer from the Γ_b_ state to the Γ_c_ state, corresponding to the metal-to-ligand charge transfer (MLCT) from the Cu(I) to the **PA** ligands (Figure 2b and Figure S4). And the strong intermolecular π–π interactions among the coordinated **PA** ligands (Figure 1b) may further extend and broaden the absorption edge [30]. Therefore, the broad-spectrum absorption of compound **1** results from the combined contributions of MLCT and intermolecular π–π interactions.

**Figure 3:**
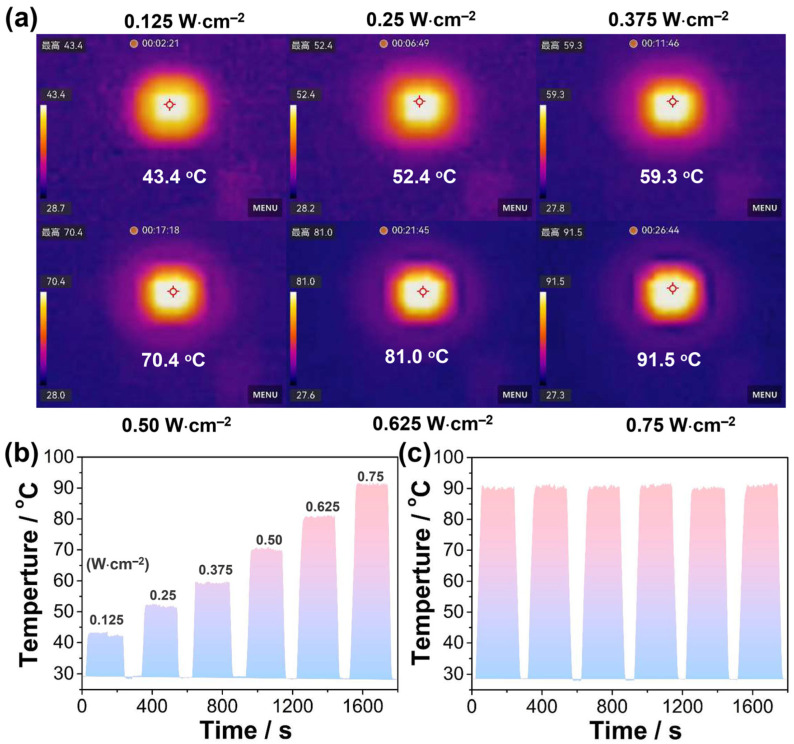
(**a**) Infrared images of a crystalline pellet of **1** under varying irradiation power densities. (**b**) Temperature variations in **1** under 808 nm laser irradiation at different power densities. (**c**) Cycling temperature profile of **1** under 808 nm laser irradiation with a constant power density of 0.75 W/cm^2^.

Encouraged by the strong absorption in the near-infrared (NIR) region and the high thermal stability of **1**, a detailed investigation into its NIR-I photothermal conversion properties was conducted. Upon irradiation with an 808 nm laser (0.75 W/cm^2^), the temperature of the crystalline pellets rapidly increased from 27.3 °C to approximately 91.5 °C (Figure S5). In contrast, no significant temperature rise was observed on a blank quartz glass plate under the same conditions (Figure S5). When the power of the 808 nm laser was varied to 0.125, 0.25, 0.375, 0.50, 0.625, and 0.75 W·cm^−2^, the corresponding temperatures of the crystalline pellets were 43.4, 52.4, 59.3, 70.4, 81.0, and 91.5 °C, respectively, demonstrating the linear response of the photothermal effects of compound **1** (Figure 3a,b). The maximum temperature changes (ΔT_max_) for these conditions were 14.7, 24.2, 31.5, 42.4, 53.4, and 64.2 K, respectively (Figure 3a,b). To assess the stability and durability of the photothermal conversion, cycling experiments were performed under an 808 nm laser with a power density of 0.75 W/cm^2^. The results demonstrated that the photothermal effects of compound **1** remained stable for at least six cycles (Figure 3c). Furthermore, based on the cooling curves and the corresponding time–lnθ linear analysis (Figure S6), the photothermal conversion efficiency (PCE, η) of compound **1** was estimated to be 50%, ranking at an upper-middle level among photothermal coordination polymer materials (Table S3). Moreover, previous studies have shown that charge transfer and intermolecular π–π interactions can lead to denser electronic energy levels in molecules, thereby facilitating the non-radiative decay of excited states through increased vibrational cooling and internal conversion. As a result, the MLCT and intermolecular π–π interactions in compound **1** are assumed to contribute significantly to its efficient photothermal conversion when it is irradiated with an 808 nm laser.

**Figure 4:**
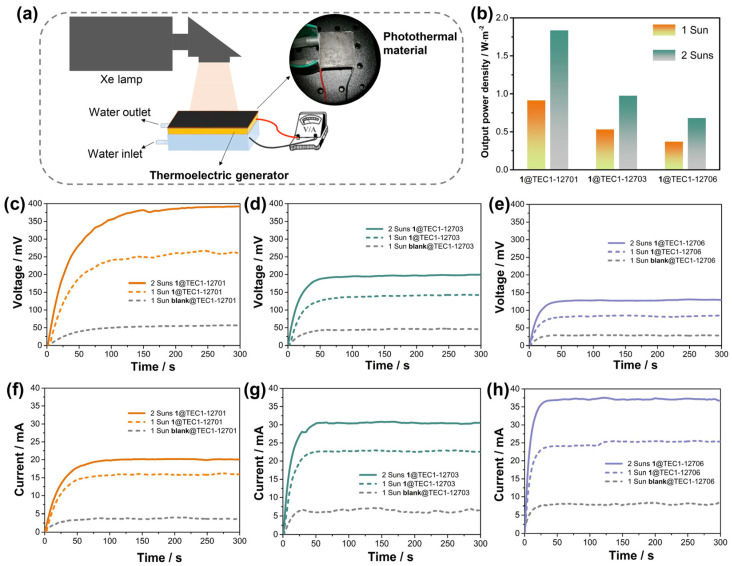
(**a**) Schematic diagram of photo−thermo−electric conversion device. (**b**) The output power density of different photo−thermo−electric conversion systems when loading external resistances under irradiation of 1 Sun and 2 Suns. Open−circuit voltages (**c**–**e**) and currents (**f**–**h**) of different commercial thermo−electric generators after loading with compound **1** under irradiation of 1 Sun and 2 Suns, respectively.

Given its broad absorption band and efficient NIR-I photothermal effect, the application of compound **1** in photo-thermo-electric conversion was investigated. An approximately 50 mg sample of crystalline powder of compound **1** was evenly applied to the surface of a commercial thermo-electric generator (TEC1-12701, Hongnuoda Technology Co., Ltd., Shenzhen, China) using thermal conductive glue (Figure 4a). Under 1 Sun irradiation provided by a solar simulator system (CEL-PF300-T9, China Education Au-light Co., Ltd., Beijing, China), the device **1**@TEC1-12701 exhibited an open-circuit voltage of 261 mV (Figure 4c) and an output current of 16.0 mA (Figure 4f). To further assess the performance, compound **1** was tested with two other commercially available thermo-electric generators, TEC1-12703 and TEC1-12706 (Hongnuoda Technology Co., Ltd., Shenzhen, China). The results showed that after coating the devices with the same weight of crystalline powder of **1**, under 1 Sun irradiation, the open-circuit voltage and current of **1**@TEC1-12703 were 142 mV and 22.5 mA (Figure 4d,g), respectively. For **1**@TEC1-12706, the open-circuit voltage and current were 86 mV and 25.4 mA (Figure 4e,h), respectively. Temperature measurements revealed negligible temperature changes of less than 1 K for the blank thermo-electric generators (Figure S7a). However, after applying the crystalline powder of **1**, the temperature differences were observed to increase, indicating that the **1**-coated layers acted as efficient photothermal materials. Although the weight of the sample applied was consistent, **1**@TEC1-12701 exhibited the largest temperature difference of 9 K (Figure S7a), highlighting the importance of the coating process in achieving high photo-thermo-electric conversion efficiency. Moreover, **1**@TEC1-12703 and **1**@TEC1-12706 were similar cases (Figure S7b,c). As reported, open-circuit voltage and current are proportional to the intensity of radiation. To improve the output performance further, the radiation intensity was increased to 2 Suns (2000 W/m^2^). Under these conditions, both the open-circuit voltage and the current were enhanced (Figure 4c–h). Notably, **1**@TEC1-12701 showed a maximum open-circuit voltage of 392 mV (Figure 4c) and a current of 20.0 mA (Figure 4f). These results suggest that a higher solar radiation power density leads to better power generation performance. The performance of **1** in this system is comparable to that of reported inorganic–organic hybrid materials, indicating that the metal halide of compound **1** holds significant potential for application in the field of photo-thermo-electric conversion.

To evaluate the maximum output power of the different thermo-electric generators coated with compound **1** under 1 Sun and 2 Sun irradiation, various external resistances were connected. The results showed that **1**@TEC1-12701 produced the highest power output when paired with an external resistance of 12.2 Ω (Figure S8), while **1**@TEC1-12703 and **1**@TEC1-12706 exhibited the peak output power at resistances of 9.3 Ω and 3.2 Ω, respectively (Figures S9 and S10). As shown in Figure 4b, **1**@TEC1-12701 demonstrated the highest output power density under both 1 Sun and 2 Sun irradiation, reaching 0.92 W/m^2^ and 1.84 W/m^2^, respectively. In comparison, under 1 Sun, **1**@TEC1-12703 and **1**@TEC1-12706 achieved maximum output power densities of 0.53 W/m^2^ and 0.37 W/m^2^ (Figure 4b), respectively. Under 2 Suns, these values increased to 0.98 W/m^2^ and 0.68 W/m^2^ (Figure 4b), respectively. Although the photo-thermo-electric conversion efficiency of **1**@TEC1-12701 is not the highest reported (Table S1), it surpasses that of the majority of photothermal materials reported to date.

## 3. Conclusions

In conclusion, this study represents the first demonstration of a photo-thermo-electric conversion application using a cuprous chloride coordination polymer. The synergy between the metal-to-ligand charge transfer (MLCT) from the Cu(I) to the **PA** ligands and the strong intermolecular π–π interactions among the coordinated **PA** ligands enables compound **1** to exhibit a broad absorption band and achieve a high NIR-I photothermal conversion efficiency (PCE) of 50% under 808 nm laser irradiation. Additionally, a photo-thermo-electric conversion device constructed using compound **1** (**1**@TEC1-12701) demonstrated a remarkable output voltage of 261 mV and an output power density of 0.92 W/m^2^ under 1 Sun (1000 W/m^2^) xenon lamp irradiation. These findings highlight the potential of cuprous halide coordination polymers as excellent NIR-I photothermal materials with broad absorption properties, developed through a charge transfer strategy. This work is expected to inspire further exploration in the fields of functional materials and photo-thermo-electric conversion technologies.

## Data Availability

The original contributions presented in this study are included in the article/Supplementary Material. Further inquiries can be directed to the corresponding authors.

## Supplementary Materials

The following supporting information can be downloaded at https://www.mdpi.com/article/10.3390/molecules29246034/s1. The general methods [31], materials, and synthesis, the computational details [32,33,34,35,36,37,38,39], and the additional table and graphics are provided in the Supporting Information. Table S1: Reported photothermal materials and their performance in photo-thermo-electric conversion when integrated with thermo-electric generators under the irradiation of 1 Sun. Table S2: Crystal data and structural refinements for compound **1**. Table S3 Reported photothermal coordination polymer materials and their photothermal efficiency under 808 nm laser irradiation. Figure S1: PXRD patterns of compound **1** and simulated data using single-crystal data. Figure S2: IR spectrum of compound **1**. Figure S3: TG curve of compound **1**. Figure S4: Partial DOS of Cu^I^ (a) and PA (b) in compound **1**. Figure S5: Temperature curves of **1** and a blank quartz glass plate under irradiation with a 0.75 W/cm^2^ 808 nm laser. Figure S6: Temperature decay curve of compound **1** after removing the laser source of 808 nm (0.75 W/cm^2^) (a) and the corresponding time–lnθ linear curve (b) [22]. Figure S7: Temperature difference (∆T) in different photo-thermo-electric conversion devices under irradiation of 1 Sun. Figure S8: Output power of **1**@TEC1-12701 under irradiation of 1 Sun (a) and 2 Suns (b) when loading different external resistances. Figure S9: Output power of **1**@TEC1-12703 under irradiation of 1 Sun (a) and 2 Suns (b) when loading different external resistances. Figure S10: Output power of **1**@TEC1-12706 under irradiation of 1 Sun (a) and 2 Suns (b) when loading different external resistances. Figure S11: Solid-state excitation spectrum monitored at 440 nm (a) and emission spectrum monitored at 330 nm (b) of **1** [40].
