# Supplementary material for: Cuprous Halide Coordination Polymer for Efficient NIR-I Photothermal Effect and Photo-Thermo-Electric Conversion
Source: Molecules. 2024 Dec 21;29(24):6034. doi: 10.3390/molecules29246034 (PMC11678656; doi:10.3390/molecules29246034)
Supplement: Supplementary file 1 [file molecules-29-06034-s001.zip › molecules-3354441-supplementary.pdf]

Supporting information

Cuprous Halide Coordination Polymer for Efficient NIR-I Photothermal Effect and Photo-Thermo-Electric Conversion

Ning-Ning Zhang*, Xiang-Tong Liu, Ke Xu, Ya-Tong Liu, Lin-Xu Liu, and Yong Yan*

School of Chemistry and Chemical Engineering, Liaocheng University, Liaocheng, Shandong 252000, P. R. China.

**Email: zhangningning@lcu.edu.cn (N. N. Zhang); yanyong@lcu.edu.cn (Y. Yan).*

1. Materials and methods

1.1 Materials

All reagents were purchased commercially and used without further purification.

1.2 Synthesis of [Cu_2Cl_2PA]$_n$ (1)

Copper(I) chloride (0.55 mmol, 74 mg), phenazine (**PA**, 0.75 mmol, 135 mg), 6 mL ethanol and 0.2 mL concentrated hydrochloric acid was transferred into a 20 mL Teflon lined autoclave and sealed. Reaction took place at 120 °C for 3 day, and black needle crystals were formed, which were collected by filtration (yield: ~75%). The phase purity of **1** was confirmed by powder X-ray diffraction (PXRD) (Figure S1) and IR (Figure S2) data. In addition, the crystal sample of compound **1** can remain stable till about 210 °C by TG analysis (Figure S3).

1.3 Characterization methods

Powder X-ray diffraction (PXRD) patterns were collected at room temperature using a SmartLab 9 Kw diffractometer using Cu Kα radiation (λ = 1.5406 Å, 450 W). Simulated PXRD patterns generated using Mercury Version 3.5.1 software based on X-ray single crystal diffraction data. Infrared (IR) data were collected using a Nicolet iS50 FT-IR spectrophotometer, covering the range from 4000−400 cm^{-1} with air asthe baseline. Thermogravimetry (TG) analyses were performed using a STA449F5-QMS403D simultaneous thermal analyzer with Al_2O_3 crucibles under N_2 (20 mL min^{-1}) at a heating rate of 10 K min^{-1} over the range of 30–800 °C. Electronic absorption spectra were measured in the diffuse reflectance mode at room temperature on a Perkin-Elmer Lambda 900 UV/vis/NIR spectrophotometer with an integrating sphere attachment and BaSO4 as a reference. A CEL-PF300-T9 xenon lamp system was used as the light source to illuminate samples for obtaining various spectra. Single-crystal X-ray diffraction data of **1** were collected on a Bruker SMART diffractometer equipped with graphite monochromated Mo Kα radiation (λ = 0.71073 Å). The crystal structure was solved and refined using the Olex2 package [1]. Positions and atomic displacement parameters were refined by full-matrix least-squares routines against F2. All hydrogen atoms were treated with a riding model. All non-hydrogen atoms were refined

anisotropically. The crystal data and structure refinement results for **1** are summarized in Table S2. Photoluminescence spectra were recorded on a FLS1000 fluorescence spectrometer using a 450 W xenon lamp as the excitation source.

1.4 NIR-I photothermal conversion measurement

NIR-I photothermal conversion measurement was conducted by irradiating pressed pellets of crystalline **1** with a laser of 808 nm produced by Changchun New Industries Optoelectronics Tech. Co., Ltd. The laser spot diameter is about 15 mm. The temperature of samples was recorded by a HIKMICRO K20 infrared camera. The temperature detecting range of the infrared camera was set as auto. The square tablet was prepared with a tablet pressing machine. The side length of the tablet is 5 mm. The weight of the crystalline pellet is about 15.3 mg.

1.5 Photo-thermo-electric conversion measurement

The photo-thermo-electric conversion experiment was performed by combining the crystalline powder of **1** and a commercially thermoelectric device. About 50 mg crystalline powder was smeared evenly on the surface of the thermoelectric device with the help of thermal conductive glue. The solar radiation of 1 Sun (1000 W/m^2) and 2 Suns (2000 W/m^2) was simulated by a Xe lamp of CEL-PF300-T9 equipped with an attenuator. The power density of CEL-PF300-T9 was monitored by a solar power meter of TES1333. Three kinds of thermoelectric devices TEC1-12701 (size: 40×40×5.2 mm), TEC1-12703 (size: 40×40×4.4 mm) and TEC1-12706 (size: 40×40×3.8 mm) were taken as thermoelectric generators. The open circuit voltage and current was recorded by an instrument of Keithley 2450. The temperature changes were monitored by an infrared camera of HIKMICRO K20.

1.6 Computational details

1) Calculation of intermolecular interactions in **1**: The plots of the electron density (ρ), reduced density gradient ($s = 1/(2(3\pi^2)^{1/3})|\nabla\rho|/\rho^{4/3}$) were obtained by density functional theory calculations. Calculations were performed with the B3LYP [2] functional and the 6-311G (d,p) basis set, using the Gaussian 16 program [3]. The results were analyzed by Multiwfn [4].
2) The DOS and PDOS were calculated by using the CASTEP package [5] in Materials studio

8.0. The structural models for **1** was built directly from the single-crystal X-ray diffraction data. The exchange-correlation energy was described by the PBE functional within the GGA [6,7]. The norm conserving pseudopotentials were chosen to modulate the electron−ion interaction [8,9]. The plane-wave cutoff energy was set as 750 eV. The Fermi level was selected as the reference and set to 0 eV by default. The smearing width was set to 0.05 eV for DOS. Other parameters were set to default values.

2. Additional tables and graphics

Table S1 Reported photothermal materials and their performance in photo-thermo-electric conversion when integrating with thermoelectric generators under the irradiation of 1 Sun.

Items	Photo-thermo-electric conversion (under 1 Sun)		Reference
	Open circuit voltage (mV)	Maximum output power density (W/m^2)	
**Organic photothermal materials**			
TQC	122.8/427	1.31/2.21	*ACS Energy Lett.* **2023**, 8, 4179−4185.
GDPA-QCN	90-100	/	*Angew. Chem. Int. Ed.* **2022**, e202117087
DDPA-PDN	83	/	*Adv. Funct. Mater.* **2021**, 2106247
4OCSPC	124	/	*J. Mater. Chem. A.* **2021**, *9*, 24452–24459
**Inorganic-organic hybrid photothermal materials**			
$\{[BaMn(ONDI)_2(H2O)_3]\cdot H_2O\}_n$	313	0.873	*Chem. Eng. J.* **2024**, 491, 152054.
$\{[Ni_4Cl_2(ONDI)_2(bpy)_4]\cdot 2Cl\cdot 2H_2O\cdot x\mathrm{DMF}\cdot yH_2O\}_n$	250	0.53	*Chem. Eng. J.* **2024**, 499, 156059.
**$[Cu_2Cl_2PA]_n$ (1)**	**261**	**0.92**	**This work**
**Inorganic composite photothermal materials**			
NF@RGO-CNT	58-59	0.251	*Ind. Eng. Chem. Res.* **2022**, 61, 16565−16576.
Ni3S2/NF	59	0.175	*ACS Sustain. Chem. Eng.* **2020**, 8, 10833−10841.
CNT	96.35	0.4	*ACS Appl. Nano Mater.* **2021**,

foam/PVA			4, 8906−8912.
PNPG/MoS2	110	0.23	*ACS Appl. Mater. Interfaces.* **2022**, 14, 1034−1044.
3D porous CPP	19.76	0.5	*ACS Sustain. Chem. Eng.* **2021**, *9*, 4571–4582.
PCC sponge	60	0.4	*Adv. Energy Mater.* **2019**, 1900250
CNTP	100	0.24	*Energy Convers. Manag.* **2021**, 241, 114306.
MnO/C-600	177	0.77	*Chem. Eng. J.* **2023**, 451, 138534.
MC10	168.3	0.6	*Energy Environ. Mater.* **2022**, 0, 1–9.
PC-x hydrogels	165.8	0.65	*Chem. Eng. J.* **2023**, 458, 141511.
PCC-800	201	0.8	*ACS Sustain. Chem. Eng.* **2022**, 10, 16427−16439.
MoS2-x NSAs	98.2	0.749	*Energy Convers. Manag.* **2022**, 252, 115070.
MSx-CPC	110	1.087	*Small* **2022**, 18, 2201949.
T-MSMD	89	0.721	*ACS Appl. Mater. Interfaces* **2021**, 13, 4305−4315.
**Polymer composite photothermal materials**			
CP@PVA	112.9	1.04	*Sci. China Mater.* **2022**, 65, 2491–2501.
DCN-4CQA@paper	91.52	0.36	*Chem. Eur. J.* **2022**, e202104137.

Table S2. Crystal data and structural refinements for compound **1**.

	**1**
**Formula**	C6 H4 Cl Cu N
**Mr**	189.10
**Crystal size (mm^3)**	0.45*0.15*0.07
**Crystal system**	triclinic
**Space group**	P −1
a **(Å)**	3.8248(3)
b **(Å)**	8.8816(7)
c **(Å)**	9.2761(8)
α **(deg)**	111.691(4)
β **(deg)**	91.874(2)
γ **(deg)**	100.715(3)
V **($Å^3$)**	285.91(4)
D_{calcd} **(g/cm^3)**	2.196
Z	2
F(000)	186.0
**Abs coeff (mm^{-1})**	4.161
$R_1{}^a$	0.0690(883)
$\omega R_2{}^b$	0.1975(988)
**GOF on F^2**	1.079

$^aR_1 = \sum||F_o|-|F_c||/\sum|F_o|$;
$^b\omega R_2 = \{\sum\omega[(F_o)^2-(F_c)^2]^2/\sum\omega[(F_o)_2]^2\}^{1/2}$.

Table S3 Reported photothermal coordination polymer materails and their photothermal efficiency under 808 nm laser irradiation.

**Coordination polymers**	**Photothermal efficiency (808 nm)**	**Reference**
$\{[La_3(bcbp)_3(NO_3)_6][La(NO_3)_6]_{1/3}\}_n$	77 %	*Chem. Commun.*, 2020, **56**, 7399–7402.
$[Ba(ONDI)(H_2O)_2]_n$	68.6%	*Inorg. Chem.,* 2024, **63**, 22502−22511.
$[Sr(BCA)_2(H_2O)_2]_n$ (1P)	53.2%	*Inorg. Chem. Front.*, 2024, **11**, 4867–4875
II-Cs-NDI	52.6%	*Inorg. Chem. Front.*, 2022, **9**, 2568–2574
**$[Cu_2Cl_2PA]_n$ (1)**	50%	**This work**
$\{[BaMn(ONDI)_2(H_2O)_3]\cdot H_2O\}_n$	42.3%	*Chem. Eng. J.*, 2024, **491**, 152054.
$\{[Cd_2(ONDI)(ox)]\cdot 2/3(H_2O)\}_n$	38.8%	*Inorg. Chem.,* 2024, **63**, 22502−22511.
II-Rb-NDI	32.1 %	*Inorg. Chem. Front.*, 2022, **9**, 2568–2574
I-Cs-NDI	31.5 %	*Inorg. Chem. Front.*, 2022, **9**, 2568–2574
THPTS-Pb	25.5 %	*Inorg. Chem.*, 2024, **63**, 3327−3334
I-Rb-NDI	23.3 %	*Inorg. Chem. Front.*, 2022, **9**, 2568–2574
$[Ag(Py\text{-}4\text{-}CSS)]_n$	22.1%	*Inorg. Chem.,* 2019, **58**, 6601–6608
$K\text{-}NDI^{\bullet-}$	11.2 %	*Materials Today Chemistry*, 2023, **27**, 101324.

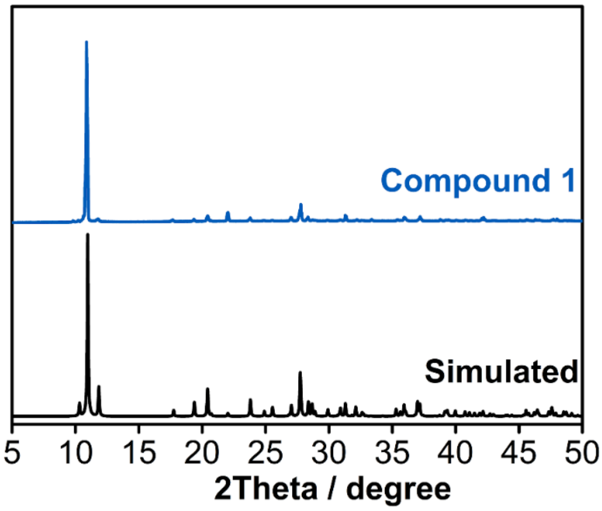

Figure S1 PXRD patterns of compound **1** and simulated data using single-crystal data.

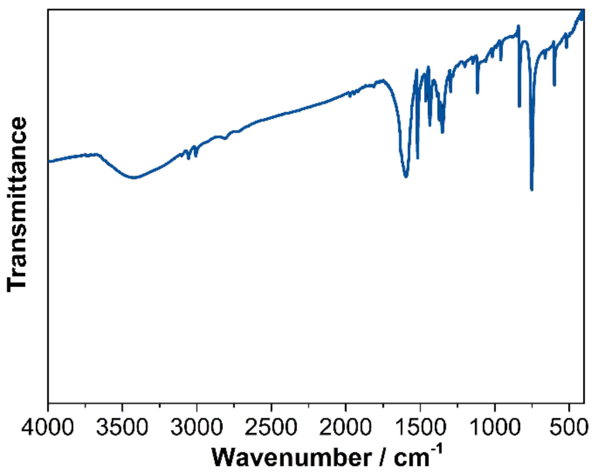

Figure S2 IR spectrum of compound **1**. This curve has not been treated by baseline deduction.

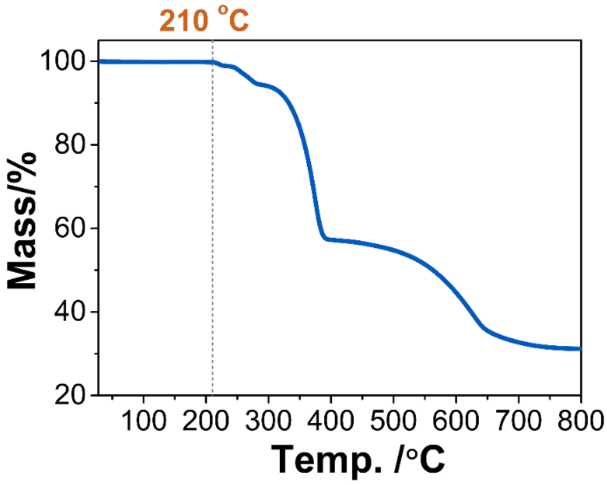

Figure S3 TG curve of compound **1**.

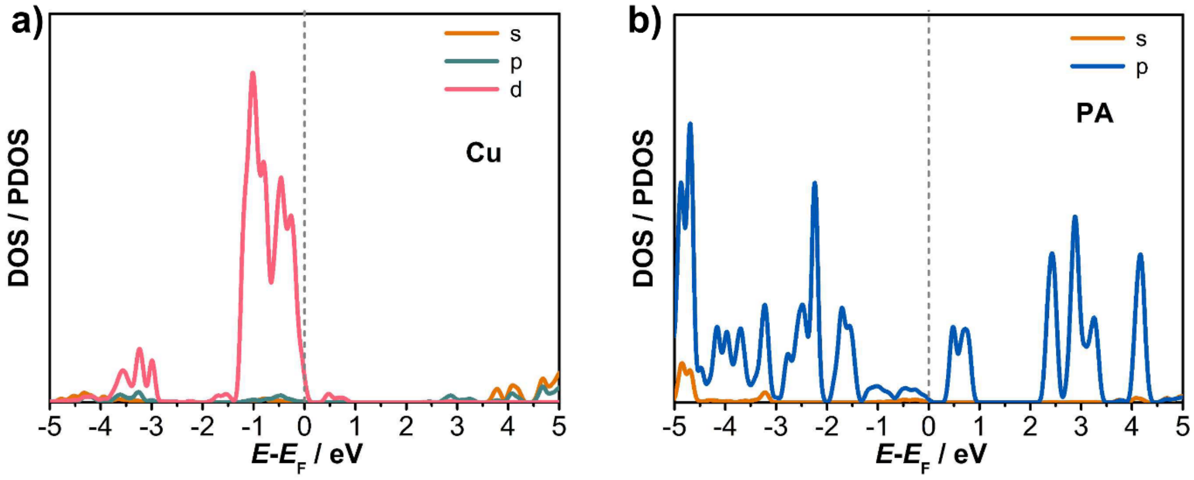

Figure S4 Partial DOS of Cu^{I} (a) and PA (b) in compound **1**.

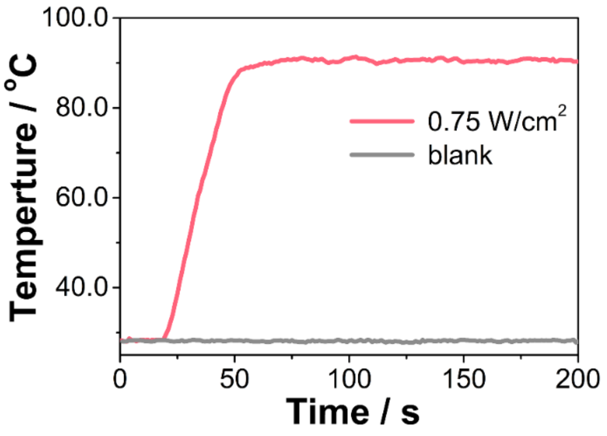

Figure S5 Temperature curves of **1** and blank quartz glass plate under the irradiation of 0.75 W/cm^2 808 nm laser.

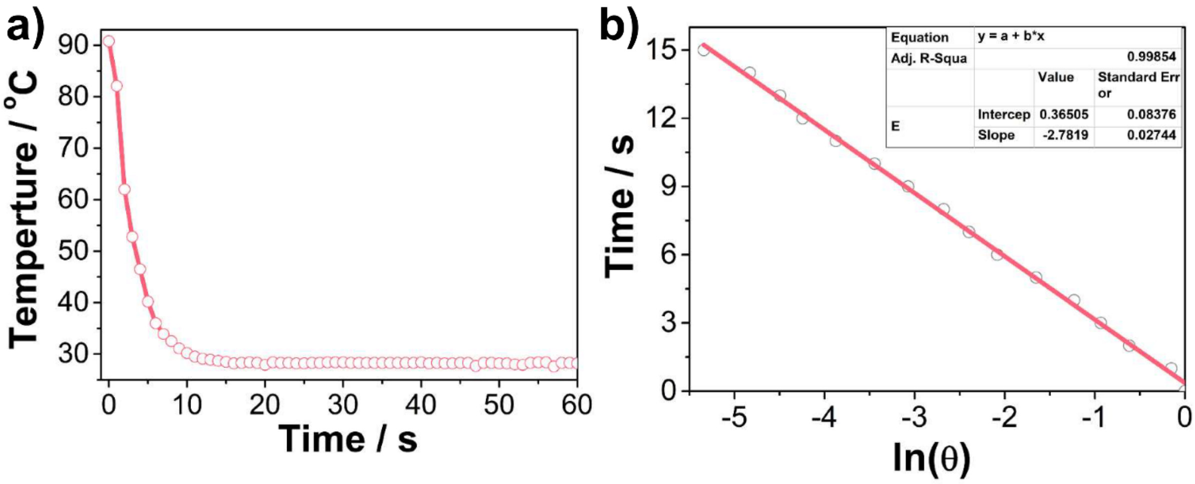

Figure S6 Temperature decaying curve of compound **1** after removing the laser source of 808 nm (0.75 W/cm^2) (a) and the corresponding time-lnθ linear curve (b). The photothermal conversion efficiency (η_{808} = 50 %) was also calculated based on reported method [10]: $\eta_{808} = \frac{hS\Delta T_{max}}{I(1-10^{-A_{808}})}$, where the I is the laser power (0.75 W/cm^2), A_{808} is the absorbance of the samples at the wavelength of 808 nm (0.54, F(R)), and ΔT_{max} is the maximum temperature change (64.2 K). hs can be calculated based on the formula of $\tau_s = \frac{\sum_i m_i C_{p,i}}{hs}$, where τ_s is the sample system time constant, m_i (0.0153 g) and $C_{p,i}$ (0.755 J·(g·°C)$^{-1}$) are the mass and heat capacity of system components. When the laser turns off, τ_s can be estimated according to the formula: $t = -\tau_s \ln\theta$. The θ can be obtained according to the formula: $\theta = \frac{T-T_{surr}}{T_{max}-T_{surr}}$, where T is the temperature of sample, T_{max} is the maximum system temperature, and T_{surr} is the environment temperature.

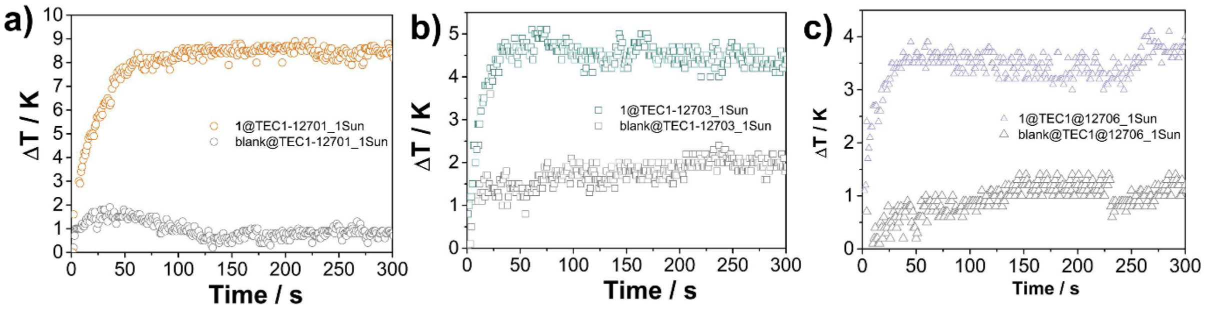

Figure S7 Temperature difference (ΔT) of different photo-thermo-electric conversion devices under the irradiation of 1 Sun.

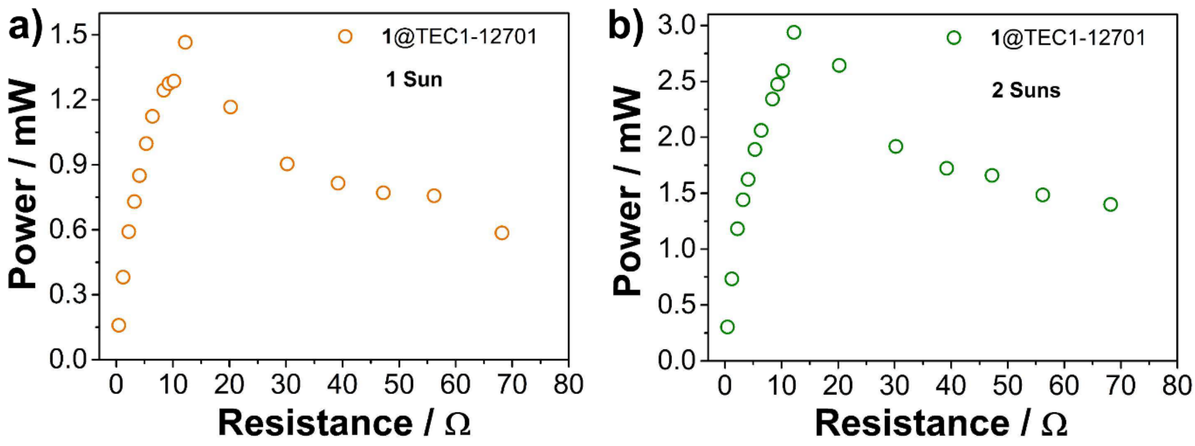

Figure S8 Output power of **1**@TEC1-12701 under irradiation of 1 Sun (a) and 2 Sun (b) when loading different external resistances.

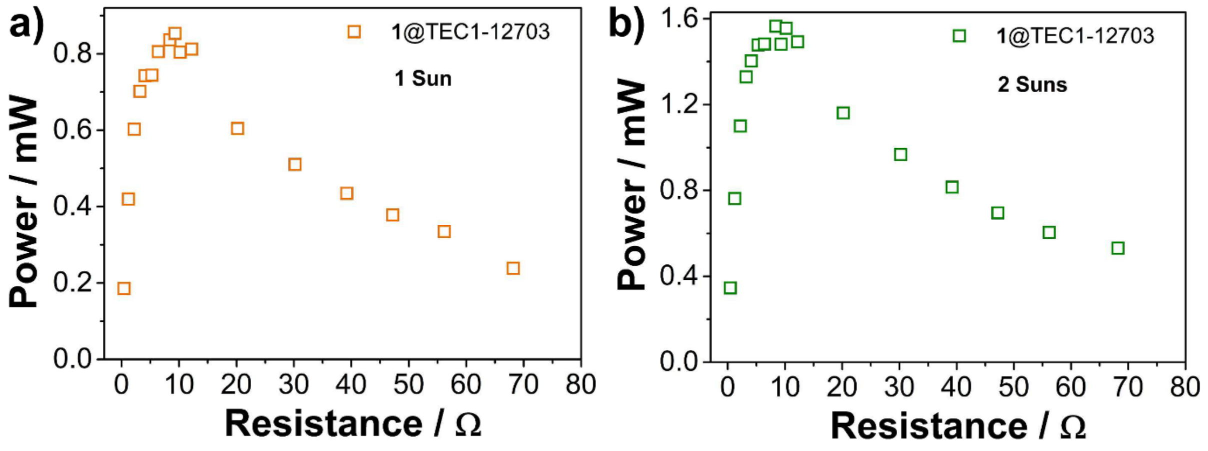

Figure S9 Output power of **1**@TEC1-12703 under irradiation of 1 Sun (a) and 2 Suns (b) when loading different external resistances.

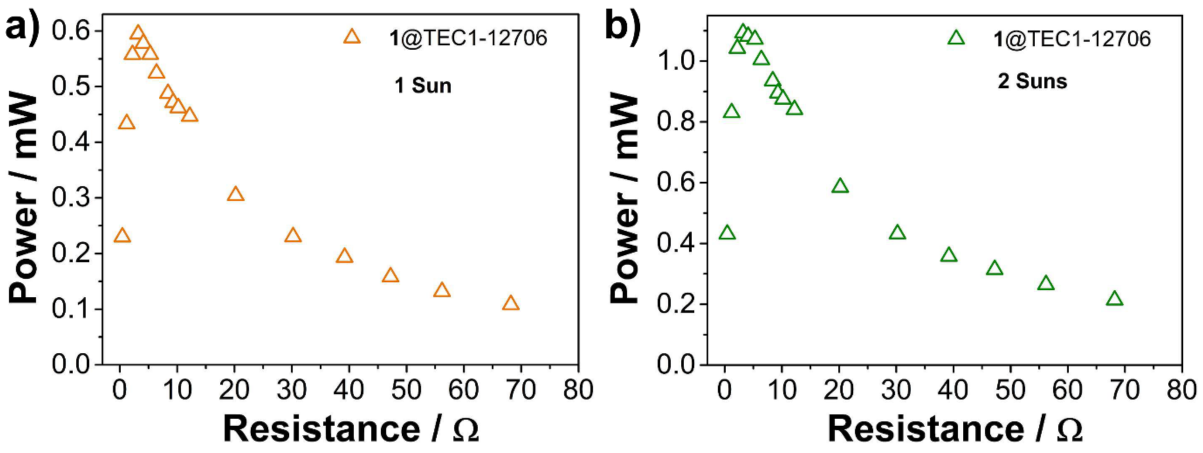

Figure S10 Output power of **1**@TEC1-12706 under irradiation of 1 Sun (a) and 2 Suns (b) when loading different external resistances.

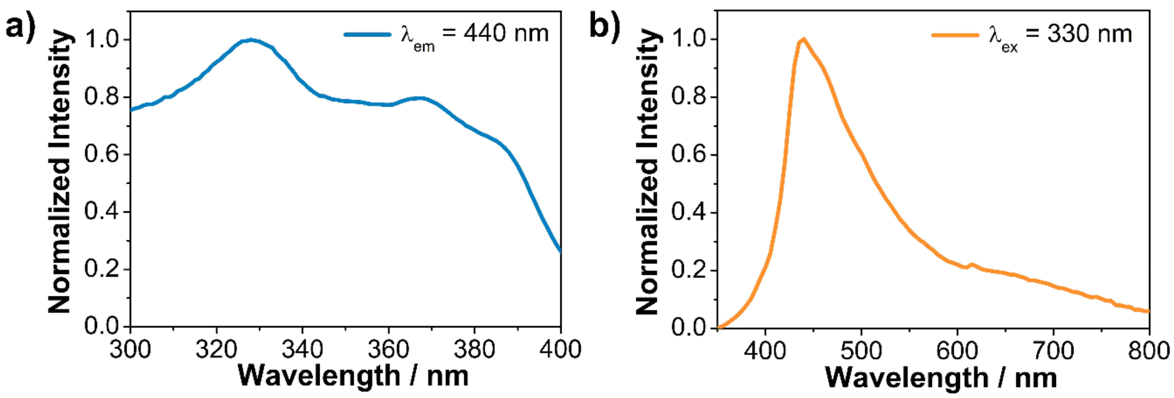

Figure S11 Solid-state excitation spectrum monitored at 440 nm (a) and emission spectrum monitored at 330 nm (b) of **1**. (Note: The emission peak of compound **1** is consistent with the emission peak of phenazine (**PA**) reported [11]. Thus, the emission source of compound **1** is the ligand (**PA**) itself.)

3. Reference

1 Dolomanov, O. V.; Bourhis, L. J.; Gildea, R. J.; Howard, J. A.; Puschmann, H. OLEX2: a complete structure solution, refinement and analysis program, *J. Appl. Crystallogr.* **2009**, *42*, 339–341.

2 Curtiss, L. A.; Redfern, P. C.; Raghavachari, K.; Pople, J. A. Gaussian-3X (G3X) theory: Use of improved geometries, zero-point energies, and Hartree–Fock basis sets. *J. Chem. Phys.* **2001**, *114*, 108−117.

3 Gaussian 16, Revision C.01, Frisch, M. J.; Trucks, G. W.; Schlegel, H. B.; Scuseria, G. E.;

Robb, M. A.; Cheeseman, J. R.; Scalmani, G.; Barone, V.; Petersson, G. A.; Nakatsuji, H.; Li, X.; Caricato, M.; Marenich, A. V.; Bloino, J.; Janesko, B. G.; Gomperts, R.; Mennucci, B.; Hratchian, H. P.; Ortiz, J. V.; Izmaylov, A. F.; Sonnenberg, J. L.; Williams-Young, D.; Ding, F.; Lipparini, F.; Egidi, F.; Goings, J.; Peng, B.; Petrone, A.; Henderson, T.; Ranasinghe, D.; Zakrzewski, V. G.; Gao, J.; Rega, N.; Zheng, G.; Liang, W.; Hada, M.; Ehara, M.; Toyota, K.; Fukuda, R.; Hasegawa, J.; Ishida, M.; Nakajima, T.; Honda, Y.; Kitao, O.; Nakai, H.; Vreven, T.; Throssell, K.; Montgomery, J. A., Jr.; Peralta, J. E.; Ogliaro, F.; Bearpark, M. J.; Heyd, J. J.; Brothers, E. N.; Kudin, K. N.; Staroverov, V. N.; Keith, T. A.; Kobayashi, R.; Normand, J.; Raghavachari, K.; Rendell, A. P.; Burant, J. C.; Iyengar, S. S.; Tomasi, J.; Cossi, M.; Millam, J. M.; Klene, M.; Adamo, C.; Cammi, R.; Ochterski, J. W.; Martin, R. L.; Morokuma, K.; Farkas, O.; Foresman, J. B.; Fox, D. J. Gaussian, Inc., Wallingford CT, **2016**.

4 Lu, T.; Chen, F. W. Multiwfn: A multifunctional wavefunction analyzer. *J. Comput. Chem.* **2012**, *33*, 580−592.

5 Clark, S. J.; Segall, M. D.; Pickard, C. J.; Hasnip, P. J.; Probert, M. I. J.; Refson, K.; Payne, M. C. First principles methods using CASTEP. *Z Kristallogr-Cryst Mater* **2005**, *220*, 567−570.

6 Hammer, B.; Hansen, L. B.; Norskov, J. K. Improved adsorption energetics within density-functional theory using revised Perdew-Burke-Ernzerhof functionals. *Phys. Rev. B Condens Matter Mater Phys.* **1999**, *59*, 7413−7421.

7 Perdew, J. P.; Wang, Y. Accurate and simple analytic representation of the electron-gas correlation energy. *Phys Rev B Condens Matter Mater Phys* **1992**, *45*, 13244−13249.

8 Hamann, D. R.; Schlüter, M.; Chiang, C. Norm-Conserving Pseudopotentials. *Phys. Rev. Lett.* **1979**, *43*, 1494−1497.

9 Lin, J. S.; Qteish, A.; Payne, M. C.; Heine, V. Optimized and transferable nonlocal separable ab initio pseudopotentials. *Phys. Rev. B Condens Matter Mater Phys.* **1993**, *47*, 4174−4180.

10 Wang, S.; Li, S.; Xiong, J.; Lin, Z.; Wei, W.; Xu, Y. Near-infrared photothermal conversion of stable radicals photoinduced from a viologen-based coordination polymer. *Chem. Commun.* **2020**, *56*, 7399–7402.

11 Wu, H.; Sun, Y.; Sun, L.; Wang, L.; Zhang, X.; Hu, W. Deep insight into the charge transfer interactions in 1,2,4,5-tetracyanobenzene-phenazine cocrystal *Chin. Chem. Lett.* **2021**, *32*, 3007–3010.
